# Fire Retardancy and Leaching Resistance of Furfurylated Pine Wood (*Pinus sylvestris* L.) Treated with Guanyl-Urea Phosphate

**DOI:** 10.3390/polym14091829

**Published:** 2022-04-29

**Authors:** Chia-Feng Lin, Olov Karlsson, Injeong Kim, Olena Myronycheva, Rhoda Afriyie Mensah, Michael Försth, Oisik Das, George I. Mantanis, Dennis Jones, Dick Sandberg

**Affiliations:** 1 Wood Science and Engineering, Department of Engineering Sciences and Mathematics, Luleå University of Technology, SE-931 77 Skelleftea, Sweden; olov.karlsson@ltu.se (O.K.); injeong.kim@ltu.se (I.K.); olena.myronycheva@ltu.se (O.M.); dennis.jones@ltu.se (D.J.); dick.sandberg@ltu.se (D.S.); 2 Structural and Fire Engineering, Department of Civil, Environmental and Natural Resources Engineering, Luleå University of Technology, SE-971 87 Lulea, Sweden; rhoda.afriyie.mensah@ltu.se (R.A.M.); michael.forsth@ltu.se (M.F.); oisik.das@ltu.se (O.D.); 3 Laboratory of Wood Science and Technology, Faculty of Forestry, Wood Sciences and Design, University of Thessaly, GR-431 00 Karditsa, Greece; mantanis@uth.gr; 4 Department of Wood Processing and Biomaterials, Faculty of Forestry and Wood Sciences, Czech University of Life Sciences Prague, Praha 6-Suchdol, CZ-16521 Prague, Czech Republic

**Keywords:** exterior wood, fire-retardant, poly(furfuryl alcohol), wood modification

## Abstract

Guanyl-urea phosphate (GUP) was introduced into furfurylated wood in order to improve fire retardancy. Modified wood was produced via vacuum-pressure impregnation of the GUP–furfuryl alcohol (FA) aqueous solution, which was then polymerized at elevated temperature. The water leaching resistance of the treated wood was tested according to European standard EN 84, while the leached water was analyzed using ultra-performance liquid chromatography (UPLC) and inductively coupled plasma–sector field mass spectrometry (ICP-SFMS). This new type of furfurylated wood was further characterized in the laboratory by evaluating its morphology and elemental composition using optical microscopy and electron microscopy coupled with energy-dispersive X-ray spectrometry (SEM-EDX). The chemical functionality was detected using infrared spectroscopy (FTIR), and the fire resistance was tested using cone calorimetry. The dimensional stability was evaluated in wet–dry soaking cycle tests, along with the mechanical properties, such as the Brinell hardness and bending strength. The fire retardancy of the modified furfurylated wood indicated that the flammability of wood can be depressed to some extent by introducing GUP. This was reflected in an observed reduction in heat release rate (HRR_2_) from 454.8 to 264.9 kW/m^2^, without a reduction in the material properties. In addition, this leaching-resistant furfurylated wood exhibited higher fire retardancy compared to conventional furfurylated wood. A potential method for producing fire-retardant treated furfurylated wood stable to water exposure has been suggested.

## 1. Introduction

The use of biomass-derived chemicals to substitute for petroleum-based products has become one of the most important topics for sustainable development [1]. Wood is a renewable biopolymer that is abundant and widely available and has been utilized for thousands of years in construction and furniture making, among other applications. Nevertheless, wood is characterized by low dimensional stability, low to medium biological resistance, and for most wood species vulnerability to fire. These drawbacks restrict the usages of wood in modern society. In this respect, several chemical modification technologies have been developed, such as acetylation, furfurylation, resin impregnation, silicate- and silane-based modification, and others [2,3,4,5,6,7,8]. Such technologies can enhance a wood’s material properties. For example, impregnation with phenol– or melamine–formaldehyde resin can increase the microbial resistance and dimensional stability of the modified wood [6,9]. Silicate- and silane-based modifications can improve the surface hydrophobicity, durability, and fire retardancy of wood [2]. However, most of the applied reagents originate from petroleum by-products or cause high levels of CO_2_ emissions [10,11,12]. Consequently, the use of more sustainable and “green“ compounds for the modification of wood should be the preferred pathway.

Furfuryl alcohol (C_5_H_6_O_2_, FA) is a liquid obtained from further treatments (dehydration and reduction) of agricultural wastes, such as sugar cane and corn cobs [13]. Using FA to modify wood has been proven to result in exceptionally high biological resistance, hardness, and dimensional stability [4,14]. The FA-treated wood (widely known as furfurylated wood) can be manufactured via vacuum pressure impregnation of porous wood species such as pine woods with an FA solution containing a stabilizer, catalyst, and co-solvents [2]. Afterwards, impregnated wood can be heated to allow the in situ polymerization of FA. This reaction basically starts with the dehydration between FA monomers at elevated temperature, with the formation of methylene bridges between the aromatic furan rings. The hydrophobic nature of the poly-FA imparts high durability to the modified wood by reducing the moisture absorption, enhancing the microbial resistance, and increasing the dimensional stability [4,15]. Nonetheless, the poly-FA itself cannot improve the fire retardancy of the furfurylated wood [16]. Very few studies have been reported on this topic to date, with the main bottleneck being the difficulty of mixing fire-retardant additives into the FA [15,17,18].

Nitrogen phosphate salts are effective fire retarding agents for solid wood and wood-based panels [17,18]. Nitrogen phosphate salts are readily available, cheap, water-soluble, and have very low toxicity. The addition of high fire retardancy to wood can be explained by several theories. Nitrogen parts are diluted by non-combustible gases through thermal decomposition into non-flammable gases such as N_2_ or NH_3_ [19,20,21]. Phosphate in the salt is extremely effective in influencing the fire retardancy, especially in the hydroxyl-rich-polymeric constituent, i.e., cellulose. This effect can be described via three different theories: (i) the released proton can accelerate the dehydration of the OH-functional groups to promote the formation of char; (ii) the dehydration of phosphate groups can release water vapor, and can also cause the formation of protective polyphosphate glassy layers to restrict contact with O_2_; (iii) the released P· radicals can act as radical scavengers to capture free radicals H· and OH· released during combustion [17,22,23,24,25]. Nitrogen-phosphate-based salts have been widely used, particularly as substitutes for highly toxic halogenated fire retardants for the fire protection of wood-based materials [26]. However, due to their high water affinity, the resulted fire-retardant treated wood products are mostly used in non-direct water contact applications, such as in indoor furniture and structures.

Hence, a simple approach to the use of nitrogen phosphate salts, without modifying their chemical formulas for exterior use, can be applied by using extra water-repellent surface protection to avoid direct contact with water. Nevertheless, the salt is hygroscopic and moisture migration during weathering would lead to the salt being eventually washed away, which would ultimately reduce the level of fire retardancy [27]. It was shown recently [28] that hydrophobic polymer matrix encapsulation can effectively enhance the water leaching resistance of the nitrogen phosphate salt by applying melamine–formaldehyde resin microspheres to encapsulate guanyl-urea phosphate (GUP) and boric acid, resulting in a highly leaching-resistant FR wood. The concept can be further expanded through using bio-derived FA instead of a synthetic polymer to produce fire-resistant wood for exterior use. Therefore, the purpose of this work was to achieve a leaching-resistant, fire-retardant treated furfurylated wood by incorporating guanyl-urea phosphate into FA solution. Our previous screening study [29] partly revealed the potential of applying the aforementioned system, although a detailed characterization and information on the influence on the wood properties are still lacking. Herein, we report on the analysis of leached water, morphology, chemical functionalities, fire performance, dimensional stability, and mechanical properties of the fire-retardant treated wood.

## 2. Materials and Methods

**Materials:** Scots pine (*Pinus sylvestris* L.) sapwood, knot- and crack-free, was obtained from Skellefteå, Sweden. Guanyl-urea phosphate (C_2_H_9_N_4_O_5_P, GUP, 98% purity) and furfuryl alcohol (C_5_H_6_O_2_, FA, 98% purity) were purchased from Fisher Scientific, Göteborg, Sweden. Triethanolamine (C_6_H_15_NO_3_, 98% purity) was bought from Sigma-Aldrich, Stockholm, Sweden. Synthesis-grade maleic anhydride (C_4_H_2_O_3_) was purchased from Merck KGaA, Darmstadt, Germany. HPLC grades of acetonitrile (C_2_H_3_N) and formic acid (CH_2_O_2_) were purchased from VWR, Stockholm, Sweden. Water was obtained using a Milli-Q Direct 8 water purification system (Merck KGaA, Darmstadt, Germany). All chemicals were used as received without purification.

**Preparation of modified wood:** Wood specimens with dimensions of 20 × 20 × 10 mm, 10 × 10 × 180 mm, and 10 × 100 × 100 mm in tangential (T) × radial (R) × longitudinal (L) directions, respectively, were conditioned at 20 °C and 65% relative humidity (RH) in air to reach an equilibrium moisture content (EMC) of approximately 12%. Their weights (m_1_) and dimensions were recorded, and the specimen volume (V_1_) values were calculated. The mean density of the conditioned specimens was 550 ± 50 kg/m^3^.

Four different formulations were prepared for wood modifications: (i) A water solution containing 30 wt% of FA and 3 wt% of maleic anhydride. The modified specimens prepared with this method were denoted as 0-30FA. (ii) A water solution containing 30 wt% of FA, 3 wt% of maleic anhydride, 3 wt% of GUP, and 1 wt% of triethanolamine was used for the modified specimens denoted as 3-30FA. (iii) A water solution containing 30 wt% of FA, 3 wt% of maleic anhydride, 5 wt% of GUP, and 1 wt% of triethanolamine. The modified specimens prepared with this method were denoted as 5-30FA. (iv) A water solution of 5 wt% GUP. The modified specimens prepared with this method were denoted as 5-0FA. The specimens were full-cell impregnated following a 30 min vacuum at 20 mbar, before 1 h of high-pressure at 15 bar. The excess quantity upon the specimen surfaces was wiped off using tissue paper, before curing in an oven at 130 °C for 24 h. The cured specimens were then conditioned at 20 °C and 65% RH and their weight (m_2_) and volume (V_2_) measurements were recorded before further experiments and analyses.

**Accelerated aging test:** The test was carried out on five replicates with dimensions of 10 × 10 × 150 mm and 10 × 100 × 100 mm (T × R × L) following the European standard EN 84 [30]. The modified specimens were conditioned at 20 °C and 65% RH, before being immersed in a 5-fold volume of deionized water in a polypropylene container. Then, vacuum was applied for 20 min in a desiccator at room temperature. The water was changed 10 times at intervals of not less than 1 day and not more than 3 days during the 14 day leaching period at room temperature. The leached wood specimens were re-conditioned at 20 °C and 65% RH before further analyses.

**Dimensional stability test:** The weight percentage gain (WPG), bulking coefficient (BC), anti-swelling efficiency (ASE), and water uptake were estimated for 5 replicates, each measuring 20 × 20 × 10 mm (T × R × L), during 4 cycles of the wet–dry test. A detailed experimental description can be found in the Supplementary Information.

**Characterization:** Ultra-performance liquid chromatography (UPLC) was performed on a Waters Acquity UPLC Class-I system coupled with a photodiode array (PDA) detector to identify the FA concentration of the leached water (i.e., leaching test according to EN 84). The maximum absorption of FA near 220 nm was used to quantify the concentration of FA, using the Waters software TargetLynx V4.2 (Milford, MA, USA). The sample was filtered with a nylon membrane 0.45 µm filter (Agilent Technologies, Santa Clara, CA, USA) before injecting 5 µL into the chromatographic column (Atlantis Premier BEH C18 AX, 1.7 µm, 2.1 × 100 mm Column, Waters, Milford, MA, USA). The elution conditions were set as follows: 0.5 mL/min flow rate; column temperature, 50 °C; autosampler temperature, 5 °C; solvent A, water with 0.1% of formic acid; solvent B, acetonitrile with 0.1% of formic. The separation gradient consisted of the following steps (expressed as % of solvent A; Supplementary Information Figure S1 shows the gradient): initially 95%; at 1 min 95%; at 5 min 80%; at 5.01 min 0%; at 7 min 0%; at 7.01 min 95%. Inductively coupled plasma-sector field mass spectrometry (ICP-SFMS) was carried out at ALS Scandinavia AB in Luleå, Sweden, to quantify the phosphorus concentration in the leached water following the EN 84 test. Water was first acidified with HNO_3_ to reach 1% HNO_3_ in water before analysis in the instrument (Thermo Scientific Finnigan MAT Element 1, Waltham, MA, USA). An optical microscope (Olympus DSX-1000 equipped with a 40× magnification lens, Tokyo, Japan) was used for the morphological investigation. For scanning electron microscopy (SEM), a Jeol JSM-IT300LV (Tokyo, Japan) equipped with an energy-dispersive X-ray spectrometer (EDX) was utilized to characterize the morphology and the elemental composition of the specimens under low-vacuum mode at 100 Pa through secondary electrons with the electron beam acceleration voltage set at 15 kV. The spectrometer was controlled through the Oxford Instrument ZAtec V3.1 software (Buckinghamshire, UK). The surfaces of the specimens were moisturized and smoothed using a rotary microtome (Leica RM2255, Nussloch, Germany) equipped with a steel blade (Leica DB80 LX, Nussloch, Germany) before subjecting it to analysis. The specimens were examined without any coating. Scanning times of about 80 s and 300 s with three replicates were used for the EDX spot analyses and EDX mapping, respectively. For the Fourier transform infrared spectroscopy (FTIR), a PerkinElmer FTIR Frontier spectrometer (Waltham, MA, USA) equipped with a UATR Diamond/ZnSe ATR (single-reflection) instrument was used to analyze the chemical functionalities of the samples. The wavenumber range of 4000–650 cm^−1^ with 4 scans at a resolution of 4 cm^−1^ was set to characterize the surfaces of the samples. Three-replicate analyses were carried out. The fire behavior was characterized using a cone calorimeter (Fire Testing Technology Ltd., East Grinstead, UK) according to ISO 5660-1 under a heat flux of 50 kW/m^2^ on three replicates with dimensions of 100 × 10 × 100 mm (T × R × L) [31]. The specimens were wrapped with aluminum foil over the bottom and sides before performing the test. At least three replicates were used. The morphology of the char residue after the cone calorimeter test was investigated by a field emission-scanning electron microscope (FE-SEM, LVEM5, Delong America, Quebec, Canada). The char residue was sputter-coated (Denton Vacuum, Desk II, Moorestown, NJ, USA) with gold at 10 nm thickness prior to backscattered electron (BSE) analysis. The beam acceleration voltage was 5 kV. A Criterion Model 43 universal testing machine (MTS Systems Corporation, Créteil, France) was used for the 3-point static bending test. The modulus of elasticity (MOE) and modulus of rupture (MOR) were measured following the standards ISO 13061-3 and ISO 13061-4, due to the smaller dimensions of the specimens [32,33]. Five replicates with dimensions of 10 × 10 × 180 mm (T × R × L) were conditioned at 20 °C and 65% RH before the measurements. The length of the span was 140 mm, and a 10 kN force cell was loaded to mid-span. The estimations used for MOE and MOR are given in Equations (1) and (2), where P is the load (N), l is the span length of the specimen (mm), b is the width of the specimen (mm), h is the height of the specimen (mm), f is the deflection (mm), and P_max_ is the maximum load (N). The Brinell hardness test was performed in a Zwick Roell ZwickiLine 2.5 TS (Ulm, Germany) universal testing machine equipped with a 2.5 kN load cell and a 10 mm diameter steel ball. The measurement followed a modified version of standard EN 1534 due to the difficulty of measuring the indentation diameter of the solid wood [34,35]. Thus, the indentation depth was measured instead. The load was gradually increased to 1 kN over 15 s and kept at 1 kN over 25 s. Then, the force was released within 15 s and the indentation depth was evaluated. The Brinell hardness was calculated following Equation (3), where F is the maximum loaded force (N), D is the diameter of the steel ball (mm), and h is the indentation depth (mm). Thirty replicates were used on each radial surface of the wood:

Modulus of elasticity (MOE) = Pl^3^/4bh^3^f (1)

Modulus of rupture (MOR) = 3P_max_l/2bh^2^ (2)

Brinell hardness (HB) = F/D*π*h (3)

## 3. Results and Discussion

**Figure 1:**
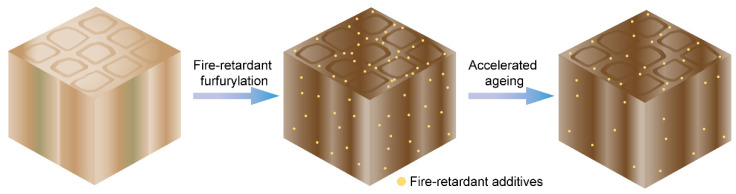
Schematic depiction of the research work.

A schematic depiction of this work is presented in Figure 1. Triethanolamine was used in this experiment as a means of stabilizing the GUP–FA aqueous solution. A phase separation can occur when extra electrolyte salt, e.g., GUP, is added to a miscible water–organic system. This typically is called the salting-out phenomenon because the hydration complex of salt leads to the phase separation from the organic compound, resulting in salt-rich and organic-rich phases [36]. The original application of the salting-out phenomenon was to purify chemicals from bio-based resources [37,38]. However, the phenomenon of salting-out is unfavorable for wood impregnation processes because the phase separation will lead to an uneven distribution of the chemicals. Thus, a surface active agent, namely triethanolamine, was utilized in this work to reduce the interfacial surface tension of the aqueous solution to prevent the phase separation [39]. Additionally, triethanolamine can also reduce undesired competing reactions, i.e., the formation of levulinic acid through the ring-opening of a furan ring under acidic humid conditions [40,41]. Figure S2 shows that a stable solution was successfully prepared with additional triethanolamine. The solution exhibited no phase separation, which can be achieved after a long storage time at room temperature.

### 3.1. Analysis of Leached Water from Treated Wood

**Figure 2:**
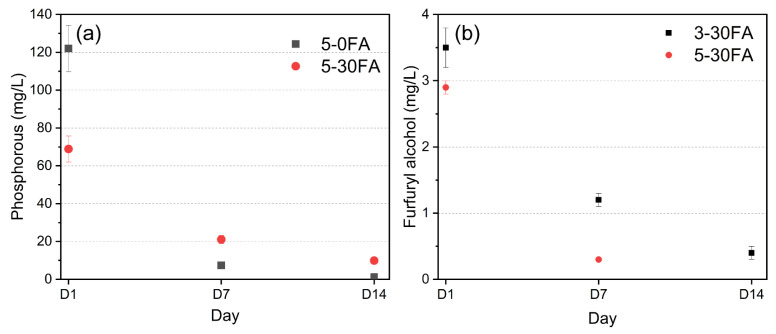
(**a**) Phosphorus concentrations of 5-30FA and 5-0FA in leached water solutions at days 1, 7, and 14. (**b**) Furfuryl alcohol concentrations in the leached water samples from 0-30FA, 3-30FA, and 5-30FA. Day 1, 7, and 14 samples from 0-30FA and day 14 sample from 5-30FA were below the detection limit (<0.1 mg/L).

For treated wood to be suitable for exterior uses, it is important to immobilize the impregnated chemicals within the wood structure to prevent them being washed away by outdoor exposure. Therefore, inductively coupled plasma–atomic emission spectroscopy (ICP-SFMS) and ultra-performance liquid chromatography (UPLC) were utilized to quantify the concentrations of phosphorus and FA in the collected water from leaching tests (EN 84). The phosphorus element in leached water was mostly derived from GUP as the unmodified Scots pine or furfurylated wood contributed to a negligible amount of phosphorus, as shown in Table S1. The phosphorus concentrations of the leached water collected at day 1, 7, and 14 for 5-0FA and 5-30FA are shown in Figure 2a. The results proved the importance of incorporating FA to reduce the leachability of water-soluble GUP. A reduction of nearly 56% of the phosphorus concentration was measured in the water collected at day 1. This was because the formed hydrophobic polymeric matrix of poly-FA captures the additive GUP, alleviating its leachability [42]. Leached water samples from 5-30FA at days 7 and 14 had higher phosphorus concentrations than those from 5-0FA because most of the GUP in 5-0FA leached out at the beginning of the test, meaning a very low concentration was detected, as shown in Figure 2a.

Non-reacted FA is volatile and moderately toxic, although the polymerized FA has no smell and is almost harmless. Figure 2b shows the FA concentrations of leached water samples collected from 0-30FA, 3-30FA, and 5-30FA at days 1, 7, and 14 during the EN84 test. The conventional furfurylated wood, 0-30FA, had a negligible amount of FA in the leached water, as the FA was nearly fully reacted by forming hydrophobic poly-FA within the wood structure. This was attributed to the high penetration of the polar FA into the cell wall and in situ polymerization at elevated temperatures with the help of the catalyst, maleic anhydride [4]. These results affirmed the previous studies showing low concentrations of the non-reacted FA in water leachates and low ecotoxicity of the conventional furfurylated Scots pine wood [40,43]. The fire-retardant treated furfurylated wood specimens, 3-30FA and 5-30FA, had higher concentrations of FA in the leached water at the beginning of the test on day 1 than the ones without the fire retardant. With further leaching, the concentration of FA in the leached water gradually reduced. The greater amounts of non-reacted FA in 3-30FA and 5-30FA were attributed to the catalyzed polymerization of FA, which was influenced by the pH of the solution, since the pH increased to about 4.5 instead of 2.0 as a result of adding GUP and triethanolamine. The pH levels of the solutions are shown in Table S2. Nevertheless, the highest concentration of non-reacted FA (3.5 mg/L in leaching water) was still considered low according to the work by Pilgård et al. [43]. European beech (*Fagus sylvatica* L.) leached out constantly, totaling approximately 12 mg/L over the whole period (14 days) of the EN 84 leaching test. This was probably due to the different grafting abilities of FA between softwood and hardwood species, as the extent of the free *ortho*-position in phenolic units is higher in softwood than in hardwood lignin [44,45]. The contents of FA in leached water in unmodified and 5-0FA specimens were below the detection limit for FA over the leaching period. All HPLC chromatograms of the analyzed water for FA retention times are presented in Figure S3.

### 3.2. Morphology and Elemental Composition

**Figure 3:**
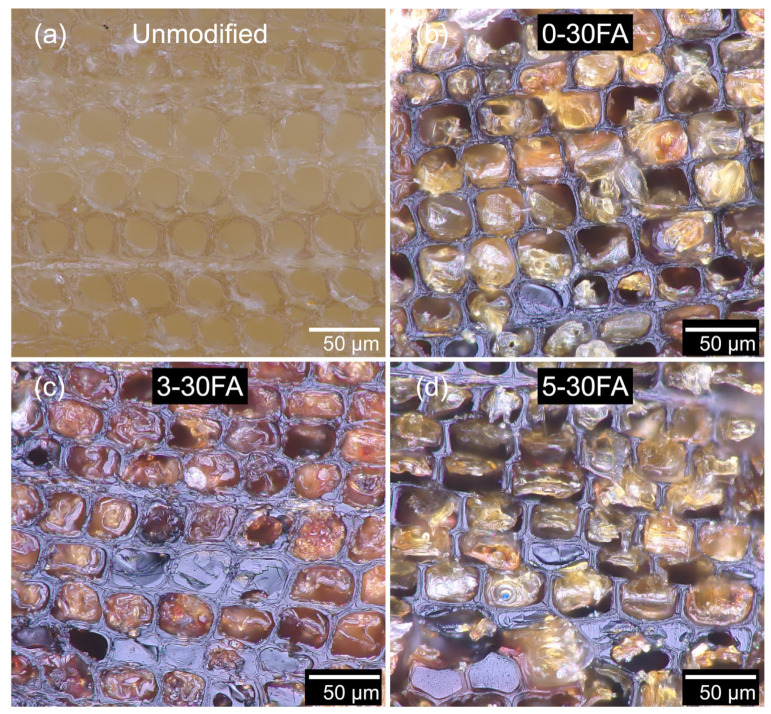
Optical microscope images of cross-sectioned (**a**) unmodified, (**b**) 0-30FA, (**c**) 3-30FA, and (**d**) 5-30FA earlywood specimens.

The cellular structure of the cross-section of Scots pine sapwood as observed using an optical microscope is presented in Figure 3a. The cellular structure and connecting pits are important for establishing deep penetration during bulk wood modifications. During the vacuum pressure impregnation, the majority of the liquid was transported along the longitudinal direction, shown as the big hollow pore between the cell wall (lumen) in Figure 3a. A minority of liquid was transported along the tangential and radial directions, in which half-bordered pits between parenchyma and bordered pits between tracheids and resin canals were responsible for liquid transportation. The furfurylated wood shown in Figure 3b had brownish cell wall and some filled-up lumens. The color change of the cell wall occurred high penetration levels of the highly polar FA and its subsequent in situ polymerization forming the conjugated poly-FA within the cell wall. The polymerization of FA starts with a dimerization reaction with the removal of a water molecule or a formaldehyde molecule before forming oligomers with sequences of furan rings linked by stable methylene bridges (conjugated structure). The oligomers can further cross-link and form branched polymers [46]. This further suggests that diffusion of the FA into the cell walls followed by in situ polymerization can result in cell wall reinforcement [47]. The FA that does not penetrate in the cell wall would remain in the lumen and polymerize there. The introduction of GUP to furfurylation showed negligible influence on the morphology of the furfurylated wood, as shown in Figure 3c,d. The images showed that the cell wall became brownish and some lumens were filled-up by poly-FA.

**Figure 4:**
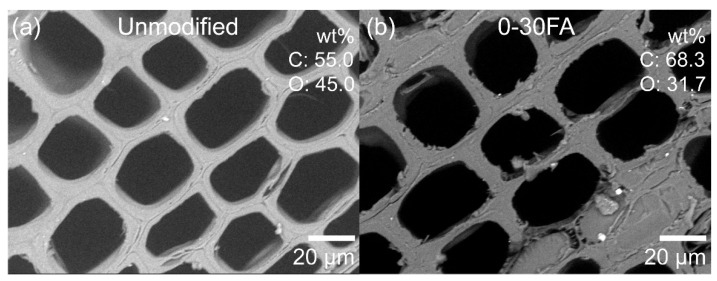
Low-vacuum SEM images and elemental composition analyzed by EDX mapping of cross-sectioned earlywood samples: (**a**) unmodified and (**b**) modified specimens (0-30FA).

To investigate the distribution of elements within the specimens, energy-dispersive X-ray spectrometry (EDX) was used. The EDX analysis confirmed that elemental C and O were detected for the unmodified wood, which was mainly attributed to the wood main components cellulose, hemicelluloses, and lignin (Figure 4a). The EDX mapping of the furfurylated wood showed that the C/O ratio increased due to the introduction of carbon-rich poly-FA (−C_5_H_4_O_1_−)_n_, as shown in Figure 4b. The higher C/O ratio and cell wall reinforcement resulted in furfurylated wood having a greater amount of char residue at elevated temperature, less water uptake, and improved dimensional stability, as discussed in the following Section 3.4 and Section 3.5.

**Figure 5:**
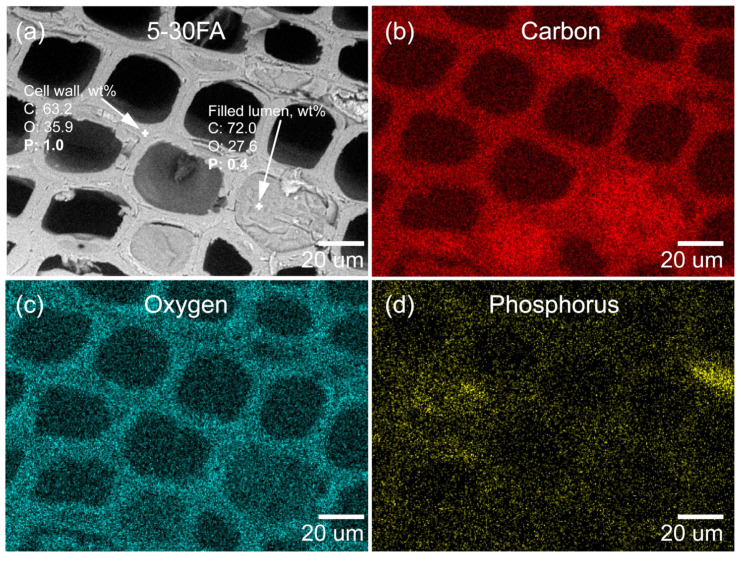
Low-vacuum SEM image and EDX spot analysis of (**a**) cross-sectioned earlywood sample (5-30FA), with corresponding EDX mapping of (**b**) carbon, (**c**) oxygen, and (**d**) phosphorus.

**Figure 6:**
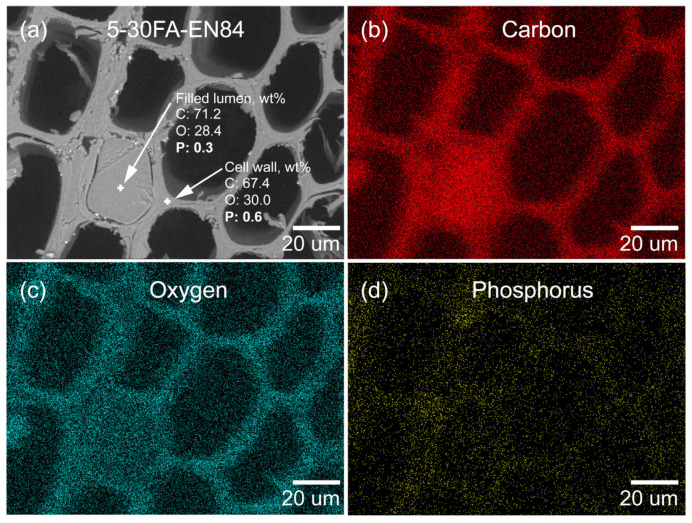
Low-vacuum SEM image and EDX spot analys-s of (**a**) cross-sectioned earlywood leached 5-30FA and corresponding EDX mapping of (**b**) carbon, (**c**) oxygen, and (**d**) phosphorus.

An EDX analysis of 5-30FA is presented in Figure 5a–d. The elemental C and O were mainly from the wood components and poly-FA. The elemental P was mainly from GUP. To further confirm the distribution of elemental P, an EDX spot analysis was performed. The results showed that GUP existed both within the cell wall as well as in the poly-FA-filled lumen, which further implied that GUP penetrated simultaneously with FA into the cell walls and could form a composite with poly-FA. Moreover, the water-leached 5-30FA was also subjected to EDX analysis to examine the existence of GUP, as shown in Figure 6a–d. The presented EDX spot analysis showed that the GUP still existed both within the cell wall and in the poly-FA filled lumen. The wt% of elemental P in the cell wall was reduced more than the poly-FA-filled lumen after the leaching test (1.0 to 0.6 in the cell wall, 0.4 to 0.3 in the poly-FA-filled lumen). This was possible because the wood components, i.e., cellulose, hemicelullose, and lignin, in the cell wall hindered the in situ polymerization of FA [47]. Conesequenlty, the lower molecular weight of FA in the cell wall led to the reduced ability for entrapping the water-soluble additive i.e., GUP. The result suggested that GUP was immobilized by the formation of the hydrophobic poly-FA matrix.

### 3.3. Chemical Functionalities

**Figure 7:**
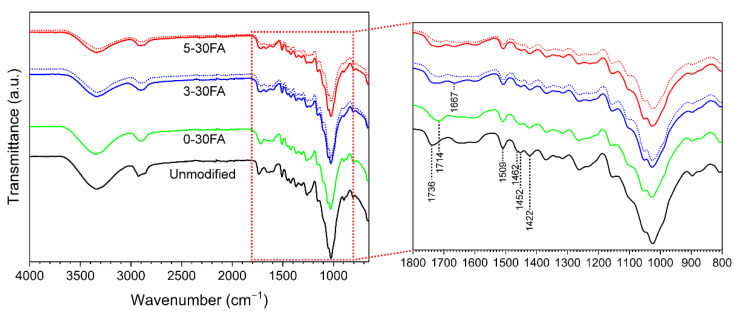
FTIR spectra of the unmodified specimen and 0-30FA, 3-30FA, and 5-30FA modified specimens. The solid curves represent pre−leaching, while the dotted curves represent post−leaching.

The chemical functionalities of the specimens analyzed by FTIR are shown in Figure 7. The spectrum of the unmodified wood was assigned to the typical wood components cellulose, hemicellulose, and lignin [28]. The bands in the region of 3000 to 2800 cm^−1^ originated from C–H aliphatic stretching of the wood (maxima at 2923 cm^−1^ and 2857 cm^−1^). The bands became one broad band at around 2900 cm^−1^ in 0-30FA due to the incorporation of poly-FA and presence of saturated C–H stretching of the methylene bridge, furan rings, and methylol groups [48]. The unconjugated C=O at 1714 cm^−1^ was related to the γ-diketones formed from the hydrolytic opening of furan rings along the poly-FA chain [49]. The bands assigned to the lignin C=C aromatic skeletal vibration at 1509 cm^−1^ and C–H vibration at 1462, 1452, and 1422 cm^−1^ showed no significant peak shifting after furfurylation [50,51]. This might imply no substantial covalent bond formation between lignin and FA, as previously observed from studies on lignin model compounds [44,52]. As the number of methylene bridges between poly-FA is likely to be considerably higher than between FA and lignin in furfurylated wood, further detailed studies are needed to confirm their existence.

In the fire-retardant furfurylated wood products, 3-30FA and 5-30FA, a new band at 1667 cm^−1^ assigned to the C=O groups from GUP was found [28]. The band at 1714 cm^−1^ became less visible and was probably because triethanolamine saponified the acids and inhibited the hydrolytic opening of furan rings [40,53]. The rest of the bands showed no difference compared to the conventionally furfurylated wood, indicating no reaction between FA and GUP. The water-leached specimens (dotted curves in Figure 7) had a less visible band at 1667 cm^−1^, which was due to the partial loss of GUP during the water leaching test. The residual bands of the water-leached, fire-retardant furfurylated wood were similar to those of the furfurylated wood.

### 3.4. Fire Performance

**Figure 8:**
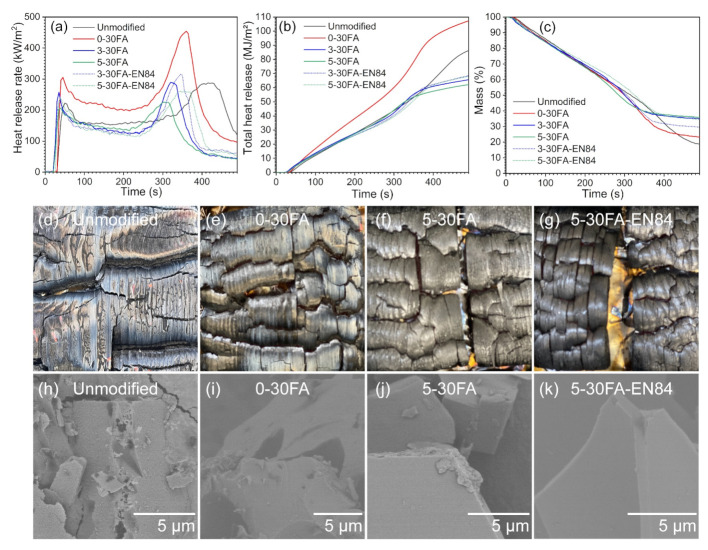
Cone calorimeter results showing (**a**) heat release rate (HRR), (**b**) total heat release (THR), and (**c**) mass values, as well as (**d**–**g**) digital photos and (**h**–**k**) SEM images of the char residues.

Cone calorimeters can be used to analyze the reaction-to-fire performance of a material by providing important parameters such as the heat release rate (HRR), peak heat release rate (pHRR), total heat release (THR), and time to ignition (TTI) [54]. The typical HRR curve of the unmodified wood showed a valley between the two peaks, as shown in Figure 8a. The first peak (pHRR_1_) corresponded to the oxidation of the wood with heat release and char formation on the surface. The reduced HRR after pHRR_1_ was due to the char acting as an insulation layer and lowering the heat release. When the fire continued burning the surface, the second peak (pHRR_2_) was observed. This might have been caused by the formation of cracks in the char increasing the sample porosity (i.e., O_2_ activity). Consequently, the fire burned into the inner part of the wood accompanied by heat release. Then, the HRR was reduced due to non-flame burning (glowing embers) after the formed volatile products were consumed [18]. The conventional furfurylated wood sample, namely 0-30FA, had higher pHRR_1_ and pHRR_2_ values compared to the unmodified wood. Additionally, the sample’s pHRR_2_ appeared within a shorter time and the total heat release (THR) was higher than for the unmodified wood. This may have been related to the exothermic reaction from the chain scission of the methylene bridge in poly-FA with the release of smaller compounds such as 2-methylfuran, 2-furfuryl-5-methylfuran, and 2,5-dimethylfuran [16,55]. However, the introduction of GUP to some extent reduced the HRR and THR compared to the unmodified sample, demonstrating GUP to be an effective fire-retardant additive [56].

Apart from the heat release, the mass residues of the wood specimens during the test were also recorded, as shown in Figure 8c. The conventional furfurylated wood had a higher mass residue after the test than the unmodified wood. This was because carbon-rich poly-FA was introduced and resulted in a higher carbon residue value. The introduction of GUP resulted in increasing mass residue values for 3-30FA and 5-30FA due to the aforementioned char promotion and the protective layer formation effects of GUP [18,29,57,58]. The furfurylated wood and fire-retardant furfurylated wood showed no significant influences on the latency of burning, as shown by the time to ignition (TTI) values in Table 1.

To examine the fire performance of the water-leached specimens, water-leached 3-30FA and 5-30FA were also analyzed. The HRR, THR, and mass loss results are presented as dotted curves in Figure 8a–c. The pHRR_1_, pHRR_2_, and THR results were slightly increased due to the partial loss of GUP. The results confirmed the ICP-SFMS, SEM-EDX, and FTIR observations. The partial loss of GUP was also reflected in the lower mass residues after combustion. Interestingly, the TTI results for water-leached 3-30FA and 5-30FA specimens were increased, which was probably due to the small organic compounds such as non-reacted FA, maleic acid, furfural, 2-furoic acid, 5-hydroxymethylfurfural (HMF), and 2,5-furandimethanol (BHMF) being washed away during the leaching test [43]. The fire growth rate (FIGRA) is calculated by dividing the maximum HRR (usually pHRR_2_ for wood material) by the time to peak heat release rate [59]. This value is used to estimate the flashover time for fire safety, as shown in Table 1. A lower FIGRA value indicates better fire performance. The conventional furfurylated wood increased the FIGRA. The introduction of GUP reduced the FIGRA value of the furfurylated wood. However, the FIGRA value of 5-30FA was slightly higher than that of unmodified wood, even though the pHHR_2_ value was reduced, which was due to the shorter time to pHHR_2_. The leached fire-retardant furfurylated wood had a slightly reduced FIGRA value, which was probably due to the mentioned small organic compounds being washed away in the specimens. To further comprehend the improvements in fire retardancy, a dimensionless criterion, the flame retardancy index (FRI), was calculated and the results are reported in Table 1. In principle, the FRI combines three important parameters, namely pHHR_2_, THR, and TTI, into one criterion [60]. The detailed FRI calculation is described in the Supplementary Information. The FRI can be used to classify a fire-retardant treatment as ‘poor’ (FRI < 1), ‘good’ (1 < FRI < 100), or ‘excellent’ (FRI > 100). The results in Table 1 show that GUP improved the fire performance of the furfurylated wood to a ‘good’ level. The water-leached specimens also exhibited ‘good’ improvements.

The digital photos and SEM images of the char residue after cone calorimetry are presented in Figure 8d–k. The SEM images of the unmodified and 0-30FA showed holes in the char residue, which could allow the ambient O_2_ to enter the underlying wood, whereas the fuel in the form of combustible gases was able to escape from this porous char layer. This consequently reduced the fire resistance. On the contrary, the introduction of GUP promoted the formation of a dense char layer with a compact microstructure that imparted greater fire resistance.

### 3.5. Dimensional Stability

**Figure 9:**
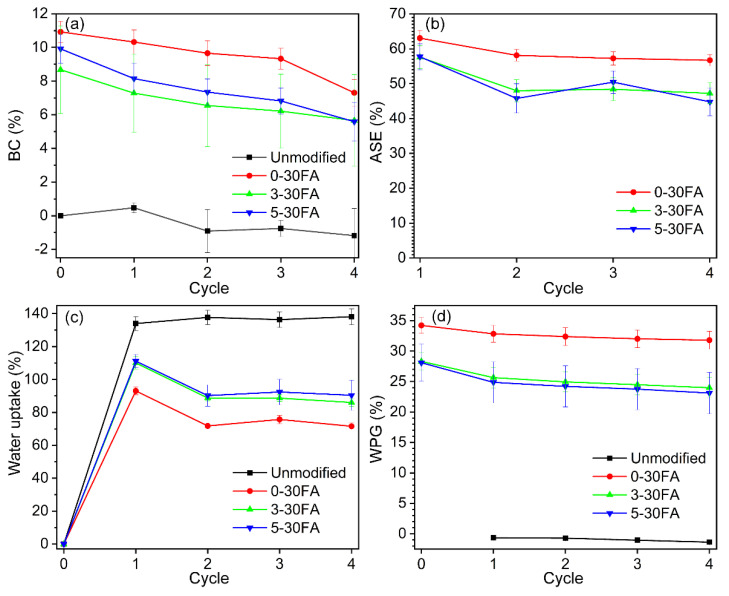
Performance of fire-retardant treated furfurylated sapwood of Scots pine: (**a**) bulking coefficient (BC), (**b**) anti−swelling efficiency (ASE), (**c**) water uptake, and (**d**) weight percentage gain (WPG) of unmodified specimen and of 0-30FA, 3-30FA, and 5-30FA modified specimens over the wet–dry cycle test.

The hygroscopic nature of the natural wood leads to dimensional swelling or shrinkage upon exposure to environmental humidity. This affects how the wood moves and distorts under dramatic humidity change scenarios, i.e., when wood is directly exposed to rain followed by sun [61]. Therefore, the repeated water saturation and drying test was selected to investigate the dimensional stability of the materials by comparing their bulking coefficient (BC), anti-swelling efficiency (ASE), water uptake, and weight percentage gain (WPG) values over the testing period. One way to achieve highly dimensional stabilized wood is by modifying the cell wall by replacing moisture absorption sites with other molecules that increase the dimensions (bulking) and reduce the dimensional instability caused by moisture sorption. The chemical used to bulk cell walls should also be immobilized during the wood modification process, thereby preventing loss of the wood’s dimensional stability during outdoor exposure [62]. Furfuryl alcohol easily penetrated in the cell walls, and high BC rates of up to 11% after the modification were achieved, as shown in Figure 9a. The leaching of non-reacted FA and other small molecules led to slight reductions in BC to 7% over the 4 cycles of the wet–dry test [43]. The introduction of a fire retardant to the furfurylated wood specimens in test groups 3-30FA and 5-30FA resulted in slightly greater BC losses after the first wet–dry cycle test. Overall, BC was relatively stable over the series of wet–dry cycle tests, while introducing GUP did not significantly change the cell wall bulking effect.

Since the partial microvoid within the cell wall was bulked-up by the in-situ-polymerized FA, the movement of the wood components was restrained [52]. The conventional furfurylated wood showed reductions of up to 60% and 40% for ASE and water uptake, respectively, as shown in Figure 9b,c. The ASE and water uptake remained relatively stable over the wet–dry cycle test. The incorporation of GUP to the furfurylated wood slightly reduced the ASE and increased the water uptake, which was probably due to the hygroscopic GUP absorbing extra water during the test.

The WPG values showed the amounts of loaded chemicals within the wood structures after modification (Figure 9d). The unmodified wood showed a slightly reduced WPG value, which was due to the removal of water-soluble extracts [63]. The small WPG loss for the conventional furfurylated wood in the first wet–dry cycle was caused by the leaching of small molecules, i.e., maleic acid, furfural, FA, 2-furoic acid, 5-hydroxymethylfurfural (HMF), and 2,5-furandimethanol (BHMF), as found in an earlier report [43]. After the first wet–dry cycle, the WPG was relatively stable. With the incorporation of GUP into the furfurylated wood, the greater WPG loss during the first wet–dry cycle test was due to the loss of the free GUP and the greater amount of non-reacted FA (Figure 2b). The WPG was relatively stable over the remainder of the wet–dry cycle test. Overall, the fire retardant slightly reduced the dimensional stability of the furfurylated wood; nevertheless, it still showed similar performance to the conventional furfurylated wood, i.e., the 0-30FA group.

### 3.6. Mechanical Properties

**Figure 10:**
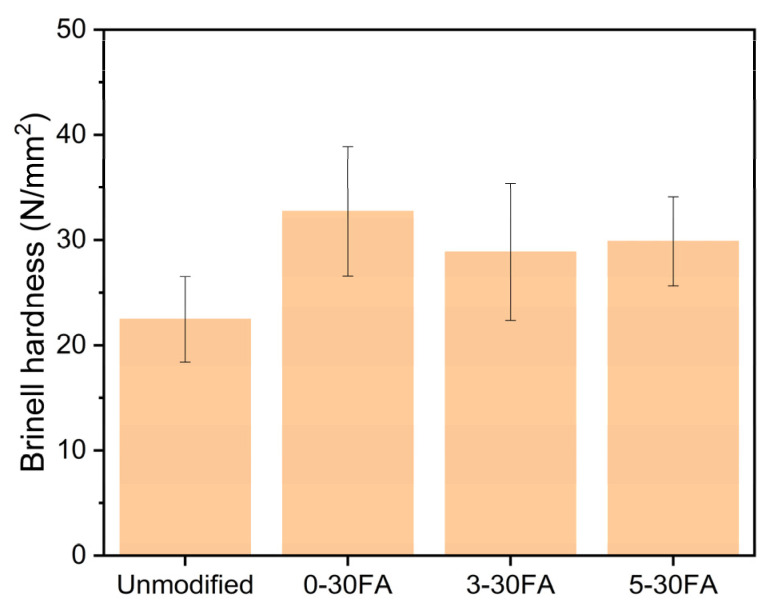
Radial surface Brinell hardness values of the unmodified wood specimen and of the 0-30FA, 3-30FA, and 5-30FA modified specimens.

The resistance of the specimen against surface deformation was measured using the Brinell hardness test, as shown in Figure 10. The conventional furfurylation (0-30FA) enhanced the Brinell hardness values due to the reinforcement of the wood cell wall and the use of poly-FA. The incorporation of GUP into furfurylated wood slightly reduced the Brinell hardness of the furfurylated wood, and this was probably because the polymerization of FA was influenced by the additives. The improved Brinell hardness was achieved due to the three-dimensional cross-linking of the linear poly-FA chain, with the addition of a sufficient amount of the acidic catalyst maleic anhydride being one of the important parameters for achieving a successful polymerization process [64,65]. The additional GUP–triethanolamine led to a less acidic solution, which probably reduced the degree of cross-linking. Thus, the hardness was not significantly enhanced.

**Figure 11:**
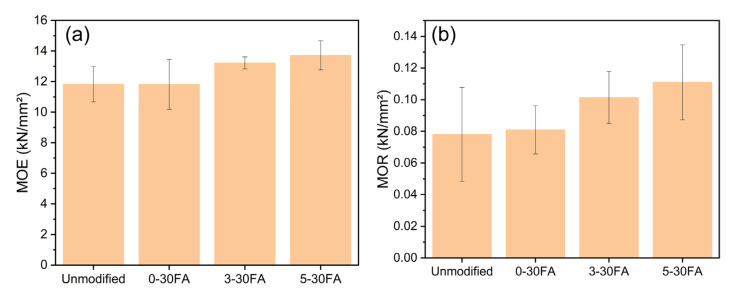
Three-point bending test to determine the (**a**) MOE and (**b**) MOR values of the unmodified wood specimen and of the 0-30FA, 3-30FA, and 5-30FA modified specimens.

The static bending related to the material’s resistance to deformation was measured via the modulus of elasticity (MOE) and modulus of rupture (MOR), with the results presented in Figure 11. The 0-30FA, 3-30FA, and 5-30FA specimens showed similar MOE and MOR values as the unmodified wood. The reinforcement of poly-FA on the wood cell walls was supposed to make the material stiffener. Nevertheless, the acidic and humid high-temperature curing conditions probably caused the thermal degradation of hemicelluloses and eliminated the reinforcement [15,66].

## 4. Conclusions

The water leaching resistance of fire-retardant treated furfurylated Scots pine sapwood was successfully achieved via in situ polymerization of FA with entrapment of the fire-retardant additive GUP. The small amount of additional triethanolamine increased the storage stability of the GUP–FA solution. The ICP-SFMS and HPLC results showed that the water leaching of GUP was alleviated by the formation of poly-FA, with no significant increase in the non-reacted FA leached into the water. The optical microscopy and SEM-EDX results suggested that GUP and FA penetrated simultaneously and were polymerized in situ to reinforce the wood cell walls. The cone calorimetry results indicated somewhat better fire performance, even after the water leaching test. The dimensional stability and mechanical properties of the fire-retardant furfurylated wood were similar to the conventionally furfurylated wood.

In summary, this work suggested a potential method for introducing additional functionality in the form of fire retardancy to furfurylated permeable softwoods stable to water leaching, and it would be interesting in future studies to investigate the performance after natural weathering or artificial weathering as the exterior wood is exposed to UV radiation.

## Figures and Tables

**Table 1:** Time to ignition (TTI), maximum heat release rate (pHRR_2_), total heat release (THR), and FIGRA values for the unmodified Scots pine specimen and 0-30FA, 3-30FA, and 5-30FA modified specimens pre- and post-leaching. The values in parentheses are standard deviations.

Specimen	Unmodified	0-30FA	3-30FA	5-30FA	3-30FA-EN84	5-30FA-EN84
TTI (s)	16.0 (5.5)	15.8 (5.7)	12.7 (0.6)	15.0 (2.6)	21.5 (1.9)	23.0 (3.6)
FIGRA (kW/m^2^s)	0.77 (0.14)	1.28 (0.08)	0.85 (0.06)	0.83 (0.15)	0.84 (0.07)	0.74 (0.00)
FRI (m^2^s/kW)	-	-	1.4	1.8	2.5	3.2
pHRR (kW/m^2^)	315.1 (40.3)	454.8 (23.7)	274.9 (24.5)	264.9 (56.8)	289.6 (27.3)	271.0 (12.8)
THR (MJ/m^2^)	79.5 (15.2)	100.4 (6.1)	66.5 (0.8)	64.8 (2.3)	69.8 (8.3)	71.2 (2.9)

## Data Availability

The data presented in this study are available on request from the corresponding author.

## Supplementary Materials

The following supporting information can be downloaded at: https://www.mdpi.com/article/10.3390/polym14091829/s1: Dimensional stability test. Figure S1: The separation gradient during HPLC. Figure S2: GUP/furfuryl alcohol solution with and without triethanolamine under room temperature. Left bottle in each figure is filled with triethanolamine; right bottle is without triethanolamine: (a) fresh prepared; (b) after 3 days; (c) after 3 weeks. The brownish color was because the polymerization of furfuryl alcohol. Figure S3: HPLC chromatogram of the leached water: (a) unmodified (b) 5-0FA (c) 0-30FA, (d) 3-30FA, and (e) 5-30FA. The retention time at 1.79 min is corresponded furfuryl alcohol. Table S1: The phosphorus concentrations of unmodified wood and modified samples according to the 0-30FA test group. Table S2: The pH values of the 0-30FA, 3-30FA, and 5-30FA solutions (pH values measured using VWR Dosatest pH test strips 0.0-6.0).
