# Supplementary material for: Fire Retardancy and Leaching Resistance of Furfurylated Pine Wood (Pinus sylvestris L.) Treated with Guanyl-Urea Phosphate
Source: Polymers (Basel). 2022 Apr 29;14(9):1829. doi: 10.3390/polym14091829 (PMC9104981; doi:10.3390/polym14091829)
Supplement: Supplementary file 1 [file polymers-14-01829-s001.zip › polymers-1663834-supplementary.pdf]

polymers

MDPI

Article

Supplementary: Fire retardancy and Leaching Resistance of Furfurylated Pine Wood (*Pinus sylvestris* L.) Treated with Guanyl-Urea Phosphate

Chia-feng Lin [1,*], Olov Karlsson [1], Injeong Kim [1], Olena Myronycheva [1], Rhoda Afriyie Mensah [2], Michael Försth [2], Oisik Das [2], George I. Mantanis [3], Dennis Jones [1,4] and Dick Sandberg [1,4]

1 Wood Science and Engineering, Department of Engineering Sciences and Mathematics, Luleå University of Technology, SE-931 77 Skellefteå, Sweden; olov.karlsson@ltu.se (O.K.); injeong.kim@ltu.se (I.K.); olena.myronycheva@ltu.se (O.M.); dennis.jones@ltu.se (D.J.); dick.sandberg@ltu.se (D.S.)
2 Structural and Fire Engineering, Department of Civil, Environmental and Natural Resources Engineering, Luleå University of Technology, SE-971 87 Luleå, Sweden; rhoda.afriyie.mensah@ltu.se (R.M.), michael.forsth@ltu.se (M.F.); oisik.das@ltu.se (O.D)
3 Laboratory of Wood Science and Technology, Faculty of Forestry, Wood Sciences and Design, University of Thessaly, GR-431 00 Karditsa, Greece; mantanis@uth.gr (G.M.)
4 Department of Wood Processing and Biomaterials, Faculty of Forestry and Wood Sciences, Czech University of Life Sciences Prague, Praha 6 - Suchdol CZ-16521, Czech Republic
* Correspondence: chia-feng.lin@ltu.se; Tel.: +46 910-585308

Dimensional stability test. Weight percentage gain (**WPG**), bulking coefficient (**BC**), anti-swelling efficiency (**ASE**), and **water uptake** were estimated for 5 replicates, each measuring 20 × 20 × 10 mm (T × R × L), during 4 cycles of the wet-dry test. In each cycle, the specimens were firstly immersed in an excess amount of deionized water before applying 30 min vacuum in a desiccator. The water was changed every 24 h and continued for 72 h. The wet weight (m_3) and volume (V_3) were recorded before subjecting to drying. The specimens were then air dried in a fume hood for 48 h before being oven-dried at 103 °C for 24 h. Weight (m_4) and volume (V_4) and were recorded after re-conditioning at 20 °C and 65% RH, before subjecting to the next cycle. WPG and BC were calculated according to Equations (S1) and (S2), respectively. The dried weight and dimension of the specimens after each cycle were also recorded to observe the WPG and BC changes (replace m_2 with m_4; V_2 with V_4). ASE was estimated according to Equation (S3), where S_1 is the swelling coefficient of unmodified wood and S_2 is the swelling coefficient of the modified wood. Swelling coefficient (S) was obtained by Equations (S4) and (S5), where V_3 is the oven-dried volume of the unmodified wood, V_4 is the water-swollen volume of the unmodified wood, V_5 is the oven-dried volume of the modified wood, and V_6 is the water-swollen volume of the modified wood. Water uptake of the specimens was calculated according to Equation (S6).

$$WPG = (m_2-m_1)/m_1 \quad (S1)$$

$$BC = (V_2-V_1)/V_1 \quad (S2)$$

$$ASE = (S_1-S_2)/S_1 \quad (S3)$$

$$S_1 = (V_4-V_3)/ V_3 \quad (S4)$$

$$S_2 = (V_6-V_5)/V_5 \quad (S5)$$

$$\text{Water uptake} = (m_3 - m_2)/m_1 \quad \text{(S6)}$$

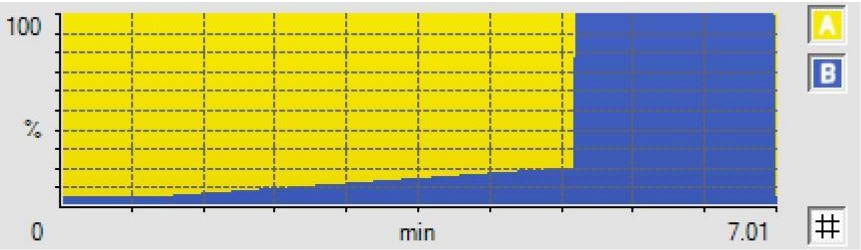

Figure S1. The separation gradient during performing HPLC.

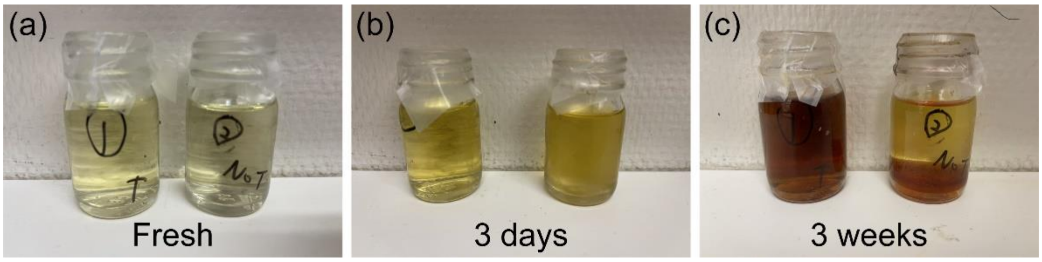

Figure S2. GUP/furfuryl alcohol solution with/without triethanolamine under room temperature. Left bottle in each figure is with triethanolamine; right bottle is without triethanolamine: (**a**) fresh prepared, (**b**) after 3 days, and (**c**) after 3 weeks. The brownish color was because the polymerization of furfuryl alcohol began.

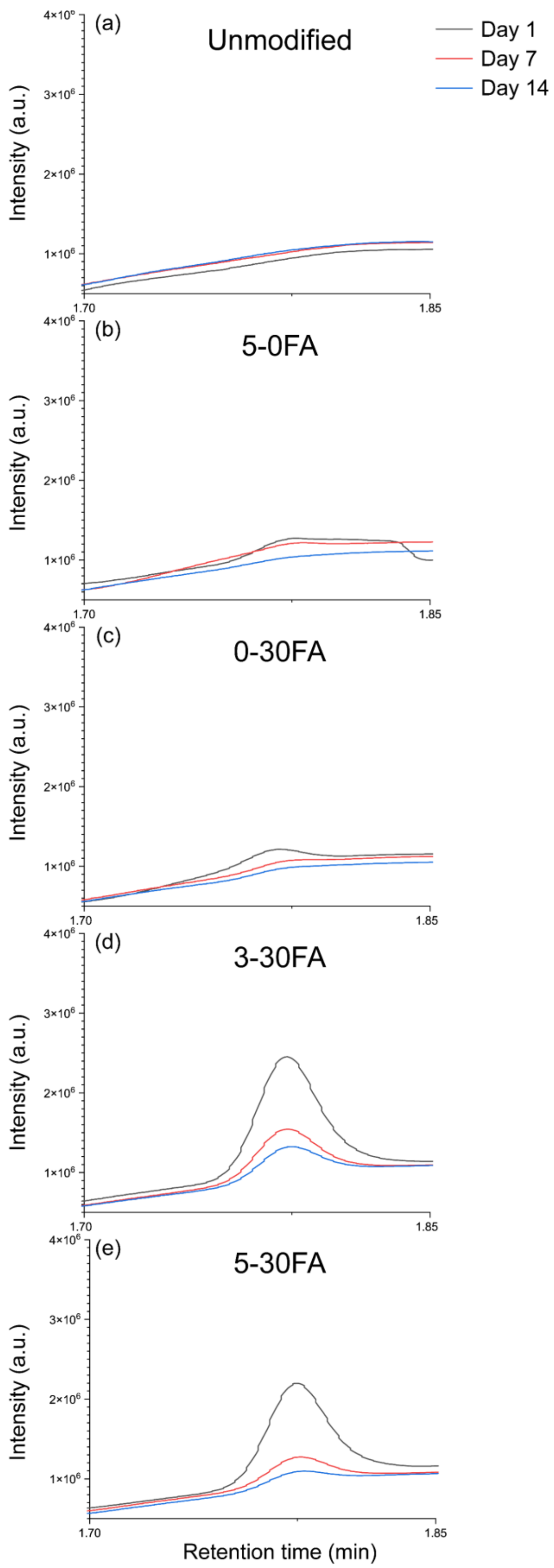

Figure S3. HPLC chromatogram of the leached water: (a) unmodified (b) 5-0FA (c) 0-30FA, (d) 3-30FA, and (e) 5-30FA. The retention time at 1.79 min is corresponded furfuryl alcohol.

Table S1. The phosphorus concentration of unmodified wood and modified according to the 0-30FA test group.

	Phosphorus concentration (mg/L)	
**Day**	**Unmodified**	**0-30FA**
1	0.118	0.050
7	<0.04	<0.04
14	<0.04	<0.04

Table S2. pH value of the 0-30FA, 3-30FA and 5-30FA solutions. (pH value was measured by VWR Dosatest pH test strips 0.0-6.0)

	**0-30FA**	**3-30FA**	**5-30FA**
pH	2.0	4.5	4.5
